# Influence of chronic liver diseases on the course and outcome of COVID-19

**DOI:** 10.1371/journal.pone.0288350

**Published:** 2023-07-14

**Authors:** Nikola Mitrovic, Milos Sabanovic, Ankica Vujovic, Jaroslava Jovanovic, Natasa Nikolic, Martina Jug, Nevena Todorovic, Ana Filipovic, Ivana Milosevic

**Affiliations:** 1 Faculty of Medicine, University of Belgrade, Belgrade, Serbia; 2 Department of Hepatology, Clinic for Infectious and Tropical Disease, University Clinical Centre of Serbia, Belgrade, Serbia; Bolu Abant İzzet Baysal University: Bolu Abant Izzet Baysal Universitesi, TURKEY

## Abstract

**Introduction:**

Coronavirus disease of 2019 (COVID-19) is a global health problem. The impact of chronic liver diseases on the course and outcome of COVID-19 is still the subject of research. The aim of this study was to show the characteristics of COVID-19 patients with chronic liver diseases, and to establish the risk factors for unfavourable outcome.

**Methods:**

A retrospective observational study was conducted at the Infectious Disease Clinic in Belgrade, Serbia, and included 80 patients with chronic liver diseases and COVID-19 within a time frame of two years (between 15 March 2020 and 15 March 2022). Characteristics of the affected persons, as well as the risk factors for a fatal outcome, were analyzed.

**Results:**

Of the 80 subjects in the study, 23.8% had chronic viral hepatitis, 12.5% autoimmune liver diseases and alcoholic liver disease respectively, 30% had non-alcoholic fatty liver disease, while 11.2% had chronic liver diseases of unknown aetiology. A total of 33.7% had cirrhosis, 6.3% hepatocellular carcinoma and 5% had liver transplants. A total of 92.5% of respondents had pneumonia (21.2% were critically ill). A deterioration of chronic liver disease was registered among 33.7% of patients, and decompensation in 3.8%; 76.3% patients recovered, while 23.7% had a lethal outcome. Risk factors for lethal outcome by univariate analysis were: alcoholic liver disease, cirrhosis, increased transaminases values prior to COVID-19, malignancy, severe pneumonia and dyspnea. In a multivariate analysis, the presence of liver cirrhosis (OR = 69.1, p = 0.001) and severe pneumonia (OR = 22.3, p = 0.006) remained independently predictive for lethal outcome.

**Conclusion:**

These findings will help with the evaluation of COVID-19 patients who have chronic liver diseases and will improve their risk stratification.

## Introduction

Coronavirus disease of 2019 (COVID-19) is a global health problem, and the pandemic has affected almost all countries of the world. According to data from the World Health Organisation (WHO), so far more than 660 million people around the world have become ill with the disease and more than 6.7 million have died [1]. COVID-19 infection is characterized with inflammation and usually presents with mild flu like symptoms [2, 3]. However, it can also progress to more serious course [4]. The clinical picture varies from mild and almost asymptomatic forms of the illness to severe, life-threatening forms accompanied by respiratory failure, sepsis, septic shock, and multiple organ dysfunction. It is an illness with the possibility of affecting different organ systems. Research so far has clearly established that the course of the illness takes a more severe form and has a poorer outcome among persons with cardiac comorbidities (cardiomyopathy, arrhythmias, coronary artery disease), chronic lung diseases (chronic obstructive lung disease, pulmonary hypertension, pulmonary embolism), neurological diseases (cerebrovascular diseases, dementia), in chronic kidney patients, and immunodeficient states (HIV, primary immunodeficiencies). As for chronic liver diseases (CLDs) and their influence on the course and outcome of COVID-19, the results are not as consistent and numerous research projects are still being conducted. Furthermore, the extent to which COVID-19 exerts an influence on CLDs has not been fully defined [5]. Prospective multicentric studies still underway indicate that persons with a terminal liver disease–cirrhosis, have a greater risk of poorer prognosis. The exact mechanism of the negative influence on respiratory tract function remains the subject of research [6]. Furthermore, it has not been fully elucidated whether the aetiology of liver diseases have an influence on the course of COVID-19 and its outcome. For instance, non-alcoholic fatty liver disease (NAFLD) and liver steatosis were initially linked to an increased risk for the severe form of COVID-19, however, it is possible that the real cause of the increased mortality is actually metabolic syndrome rather than the liver disease itself [7]. For patients with autoimmune hepatitis, the assumption is that the immunosuppression associated with treatment makes them a priority for vaccination, but further analysis from matched studies, comparing them to persons with other CLDs, showed a similar outcome. Considering these results, a reduction of immunosuppressive agents for the purpose of lowering the risk of an unfavourable outcome of COVID-19 is not recommended [8]. Similarly, definitive conclusions have not been reached about the influence of chronic viral liver diseases, such as hepatitis B and hepatitis C, on COVID-19. The results of meta-analyses and systemic overview studies were inconclusive [9].

The aim of this paper is to show the characteristics of COVID-19 patients with CLDs, and to establish the risk factors in connection with CLDs for the severe clinical picture and unfavourable outcome. We also analysed the influence of COVID-19 on the deterioration of CLDs.

## Methods

To provide an answer to the question that was posed, a retrospective observational study was conducted on subjects with a previously established diagnosis of chronic liver disease (CLD) and a confirmed case of COVID-19. They were treated at the Clinic for Infectious and Tropical Diseases, University Clinical Center of Serbia during the current COVID-19 pandemic between 15 March 2020 and 15 March 2022. All the protocols performed in this study were in accordance with the ethical guidelines of the Declaration of Helsinki. Ethical approval for this study was obtained from the Ethics Committee of the University Clinical Center of Serbia. Written informed consent was obtained from each patient included in the study. The criteria of inclusion in the study were a confirmed SARS-CoV-2 infection with viral DNA detection carried out by a PCR method using a nose and throat swab, and the presence of a CLD made at least six months prior to infection. Patients with the following liver diseases were included in the study: chronic hepatitis B, chronic hepatitis C, autoimmune liver diseases (autoimmune hepatitis, primary biliary cholangitis–PBC, primary sclerotising cholangitis–PSC), alcoholic liver disease (ALD), NAFLD, and CLDs of unknown aetiology (persons with transaminase values increased by more than a factor of 2 UNL for more than 6 months, whereby the diagnoses undertaken did not uncover the aetiology of the liver disease). In the first descriptive part of the study, we presented and analysed the subjects’ demographic data, the severity of the CLD and associated comorbidities. We then presented and analysed the clinical characteristics of COVID-19, its course and outcome. The severity of COVID-19 was analysed in comparison with the severity of the pneumonia and the need for oxygen support, by categories as follows: absence of pneumonia, mild pneumonia without the need for oxygen support, middle severe pneumonia affecting less than 50% of the lung parenchyma but with the need for oxygen support, severe pneumonia affecting more than 50% of the lung parenchyma and the need for greater oxygen support, and the critically ill who were treated in intensive care unit (ICU) and required high flow oxygen support, non-invasive or invasive mechanical ventilation. The treatment was carried out according to the National Treatment Protocol for COVID-19 Patients [10], which is in accordance with the recommendations of the European Centre for Disease Prevention and Control (ECDC) [11]. In the second part of the study, we analysed the prognostic factors for COVID-19 outcomes. We analysed how the aetiology, severity, and manifestation of CLDs influenced the clinical picture, severity, and outcome of COVID-19. These prognostic factors for the liver were also analysed in relation to the previously well-defined COVID-19 prognostic factors (demographic characteristics, constitution, clinical characteristics, and associated comorbidities), with the aim of identifying and eliminating any potential confounding variables.

### Statistical analyses

Descriptive and analytical statistical methods were used. Quantitative variables were presented as mean ± standard deviation (SD) or median value. Qualitative variables were presented as frequency and percentages (%). The Chi-squared Test and Mann-Whitney U Test were used to determine the statistical significance of certain prognostic factors. The risk factors for which the univariate analysis gave p<0.1 were included in multivariate analyses using conditional multiple logistic regression. P values <0.05 was considered to indicate statistical significance. Statistical analyses was carried out with Statistical Package for the Social Sciences (SPSS) version 23 (IBM Corp, Armonk, NY, USA).

## Results

Within the timeframe of two years 80 patients with CLD who contracted COVID-19 were included in the study; of these 59 (73.8%) were male and 21 (26.3%) were female. The average age was 55.5±14.3 years, with a range of 20–78 years. Of these, 49 (61.2%) were of average constitution (BMI 18.5–24.9 kg/m^2^), 7 (8.8%) were underweight (BMI <18.5 kg/m^2^), while 24 (30%) were overweight (BMI>25 kg/m^2^). Of the CLDs, the greatest number were with chronic hepatitis B virus (HBV) and hepatitis C virus (HCV) infections– 27, somewhat fewer were with NAFLD– 24, while other causes were less represented. A total of 27 (33.7%) had liver cirrhosis. In terms of transaminase level before the onset of COVID-19, 60% of the subjects had active disease. The characteristics of subjects in terms of liver disease are shown in Table 1. A total of 52 patients (65%) had comorbidities that would influence the severity and outcome of COVID-19. Chronic comorbidities are shown in Table 2.

**Table 1:** Characteristics of patients with regard to chronic liver disease.

Characteristics	Number of patients (%)
**Etiology of chronic liver disease**	
Chronic hepatitis B	14 (17.5%)
Chronic hepatitis C	13 (16.3%)
Autoimmune liver diseases^a^	10 (12.5%)
Alcoholic liver disease	10 (12.5%)
Non-alcoholic fatty liver disease	24 (30.0%)
Chronic liver diseases of unknown aetiology	9 (11.2%)
**Severity of the chronic liver disease**	
Absence of fibrosis or mild fibrosis	47 (58.8%)
Advanced fibrosis	2 (2.5%)
Compensated cirrhosis	8 (8.8%)
Decompensated cirrhosis	14 (17.5)
Hepatocellular carcinoma (with liver cirrhosis)	5 (6.3%)
Liver transplant	4 (5.0%)
**Presence of active liver disease** ^**b**^	
Normal values of transaminases	32 (38.8%)
Values of transaminases increased less than two-fold UNL	10 (12.5%)
Values of transaminases increased two- to three-fold UNL	12 (15.0%)
Values of transaminases increased more than three-fold UNL	26 (32.5%)

UNL, upper normal limit

^a^ autoimmune hepatitis, primary biliary cholangitis, primary sclerotising cholangitis.

^b^ in relation to the value of transaminases by regular monitoring before the onset of COVID-19.

**Table 2:** Comorbidities that have been associated with severity and outcome of COVID-19 in previous studies.

Comorbidities	Number of patients (%)
**Heart diseases**	
Hypertension	35 (43.8%)
Cardiomyopathies	9 (11.3%)
Heart arrhythmias	6 (7.5%)
**Endocrine diseases**	
Thyroid disease	5 (6.3%)
Diabetes mellitus type 2	13 (16.3%)
Insulin-dependent diabetes mellitus type 2	7 (8.8%)
**Chronic lung disease**	
Asthma	3 (3.8%)
Chronic bronchitis	4 (5.0%)
**Chronic kidney disease**	6 (7.5%)
**Malignancy**	
Carcinomas	7 (8.8%)
Hematologic malignancies	1 (1.2%)

### COVID-19

The average duration of the illness prior to hospitalisation amounted to six days (median value). Most of the patients (94.8%) had a fever, average temperature of 38.4±0.8°C. The following complaints were dominant: weakness and fatigue (63.8%) and cough (61.3%), while other complaints were less present. Most of the patients had pneumonia (92.5%), most often middle severe (40%), with average computed tomography scan (CT scan) severity score of 13.8±5.7. The clinical characteristics of the subjects are shown in Table 3.

**Table 3:** Clinical characteristics of COVID-19 patients with chronic liver diseases.

Clinical characteristics	Number of patients (%)
**Fever**	
Absence of fever	5 (6.2%)
Low-grade fever (≤37.9°C)	11 (13.8%)
Mild to moderate fever (38–39°C)	37 (46.3%)
High fever (>39°C)	27 (33.7%)
**Weakness and fatigue**	51 (63.8%)
**Anosmia and ageusia**	5 (6.3%)
**Cough**	52 (65%)
**Dyspnea (new or worsening over baseline)**	27 (33.8%)
**Vomiting and diarrhea**	10 (12.5%)
**Pneumonia due to SARS-CoV-2**	
Absence of pneumonia	6 (7.5%)
Mild pneumonia	20 (25%)
Middle severe pneumonia	32 (40%)
Severe pneumonia	5 (6.3%)
Critically ill	17 (21.2%)

Antiviral treatment was dispensed to 20 (25%) of patients, as follows: Favipiravir 15 (18.8%), Remdesivir 1 (1.3%), and Molnupiravir to 4 (5.0%). Anti-inflammatory cortical steroid treatment was dispensed to 59 (73.7%) subjects, and Tocilizumab to 27 (33.8%). Anticoagulant treatment was dispensed to 67 (83.8%), and Vitamins C and D to 68 (85%). There were 16 (20%) vaccinated and the majority had received the Sinopharm inactivated vaccine– 12 (15%). Two patients had received the Pfizer and Sputnik V vaccines.

Deterioration of the CLD was found among 27 (33.7%) with decompensation registered among 3 (3.8%), while the remainder had a spike in values of transaminases.

Full recovery was registered in 58 (72.5%), partial recovery with the need for follow-on oxygen support after discharge among 3 (3.8%), while 19 (23.7%) had a lethal outcome. The duration of the illness from onset of initial symptoms to end of illness was 20.4±8.0 days, with a range of 5–50 days.

### Analysis of risk factors

Analysing the severity of the clinical presentation of COVID-19 established that the more severe forms appeared in patients with liver cirrhosis rather than in those who did not have it. Thus, severe pneumonia and the need for treatment in ICU was registered among 40.7% of patients with liver cirrhosis as compared to 18.8% without it (p = 0.038). Analysing the aetiology of CLD in relation to the severity of COVID-19, the greatest percentage of subjects with severe pneumonia and critically ill was registered among persons with ALD (40%), but this difference was not statistically significant (p = 0.73). In relation to the activity of CLD, the more severe COVID-19 was registered among those who had elevated values of transaminases prior to COVID-19 as opposed to those who had normal values (26.7 versus 18.8 respectively), but without statistical significance (p = 0.461).

Aetiology had a significant influence on lethal outcome. Thus, a lethal outcome among subjects with infectious liver disease was registered among 22.2%, 50% with autoimmune disease, 60% with ALD, 4.2% with NAFLD and 11.1% of those with unknown cause (p = 0.002). Post-hoc analysis established a significant link between ALD and lethal outcome (p = 0.0039), which is less than the adjusted Bonferroni corrected p value (0.005). Among patients with liver cirrhosis, the outcome was lethal for 17 (63%), compared with 2 (4.1%) of those without liver cirrhosis (p<0.001). Among the subjects with liver cirrhosis who died, 11 had severe pneumonia, while the other 6 had signs of decompensated liver cirrhosis with deterioration (including three with HCC). Concerning the activity of CLD, a lethal outcome was registered among 53.3% of subjects with elevated values of transaminases before the onset of COVID-19, and among 6.3% of subjects with values of transaminases in the normal range (p = 0.005). Analysing the influence of other chronic illnesses on lethal outcome, this was more frequently registered among patients with diabetes mellitus, Hashimoto’s thyroiditis, chronic kidney diseases, and malignancies. Nevertheless, significance was found only for malignant diseases (p = 0.016).

The subjects who suffered a lethal outcome were elderly (average age of the deceased was 58.8±12.3 years, and 54.5±14.8 for those who recovered), though this age difference was not significant (p = 0.075).

In terms of complaints, only difficulty breathing was linked with lethal outcome, so that a lethal outcome was registered among 48.4% of subjects who had this symptom and among 10.2% of those who did not experience difficulty breathing (p<0.001).

As for treatment, a larger percentage of the deceased was registered among persons who did not receive antiviral treatment (25% versus 20% of those treated), but this difference was not significant (p = 0.649). Furthermore, not even the administration of cortical steroids and Tocilizumab had a significant influence on lethal outcome (23.8% versus 23.7% of those treated with corticosteroids, p = 0.994, and 22.6% versus 25.9% for Tocilizumab, p = 0.744). Lethal outcome was registered among 12.5% of vaccinated persons, contrasting with 26.6% among those unvaccinated, though this was a non-significant difference (p = 0.333). The risk factors for lethal outcome that were analysed are shown in Table 4.

**Table 4:** Risk factors for lethal outcome.

Risk factors	No. of recovered patients (%)	No. of patients with lethal outcome (%)	P-value^a^
Age ≥65 years	16 (64%)	9 (36%)	0.083
Chronic viral hepatitis	21 (77.8%)	6 (22.2%)	0.002
Autoimmune liver diseases	5 (50%)	5 (50%)	
Alcoholic liver disease	4 (40%)	6 (60%)	
Non-alcoholic fatty liver disease	23 (95.8%)	1 (4.2%)	
Chronic liver diseases of unknown aetiology	8 (88.9%)	1 (11.1%)	
Cirrhosis	10 (37%)	17 (63%)	<0.001
Increased transaminase values prior to the appearance of COVID-19	7 (46.7%)	8 (53.3%)	0.023
Severe pneumonia and critically ill	10 (47.6%)	11 (52.4%)	0.001
Diabetes mellitus	16 (80%)	4 (20%)	0.451
Heart diseases	29 (80.6%)	7 (19.4%)	0.413
Chronic lung disease	6 (85.7%)	1 (14.3%)	0.469
Chronic kidney disease	4 (66.7%)	2 (33.3%)	0.439
Malignancy	3 (37.5%)	5 (62.5%)	0.016
Cough	38 (76.9%)	11 (23.1%)	0.847
Dyspnea	14 (59.1%)	13 (48.1%)	<0.001
Weakness and fatigue	40 (78.4%)	11 (21.6%)	0.741
If antivirals were not used	45 (75%)	15 (25%)	0.649
If corticosteroids were not used	16 (76.2%)	5 (23.8%)	0.994
If Tocilizumab was not used	41 (77.4%)	12 (22.6%)	0.744
Unvaccinated subjects	47 (73.4%)	17 (26.6%)	0.333

^a^significance was calculated using the Chi-square test comparing subjects with the specified characteristic (risk factor for lethal outcome) with subjects without it

Multiple logistic regression analysis encompassed the following significant predictive factors of lethal outcome using univariate analysis: ALD, the presence of liver cirrhosis, increased transaminases prior to COVID-19, severe pneumonia, and the age of the subject (p<0.1). The following significant independent predictors of lethal outcome stood out: the presence of liver cirrhosis (the risk of lethal outcome was 69.1 times higher, p = 0.001) and severe pneumonia (a 22.3 times higher risk, p = 0.006). The remainder of the analysed variables were not significant predictors of lethal outcome in the multivariate analysis.

### Discussion

Numerous previous studies have demonstrated that mortality from COVID-19 is some 3% [4]. The following unfavourable prognostic factors for severe COVID-19 have been clearly indicated: the elderly, obesity, chronic heart and pulmonary diseases, chronic kidney diseases, immunodeficiencies and neoplastic conditions, such as CLDs [12]. Nevertheless, considering that CLD is not a single pathological entity, but rather differs based on aetiology, activity, fibrosis stage and possible liver failure, and treatment, its influence on the course and outcome of COVID-19 is not that clear [13, 14].

In this study the mortality rate of patients with COVID-19 and CLDs was 23.8%. This rate is similar to the result obtained by the International Register of Great Britain (20%), conducted by Marjot *et al*. [6]. Further comparison shows that in our study the mortality rate was lower among patients who did not have liver cirrhosis (4.3% versus 8%), while it was higher among subjects with cirrhosis (63% versus 32%). The reason for the higher mortality rate among patients with cirrhosis may lie in the fact that our study, compared with Marjot’s study, had a higher proportion of males, obese persons, the subjects had a higher rate of cardiovascular diseases and HCC, and it also included transplant patients. Each of these individual reasons could give the higher mortality rate among subjects with cirrhosis that we found [9, 12, 15]. It is speculated that patients with liver cirrhosis have a higher COVID-19 mortality rate, primarily due to a dysfunctional immune system (cirrhosis-associated immune dysfunction—CAID), which leads to greater susceptibility to infection and aberrant inflammatory response during infection. This immune dysfunction includes reduced components of the complement system, tool-like receptor upregulation, neutrophile, lymphocyte and macrophage function disorder, and intestinal dysbiosis. All of this leads to increased sensitivity to bacterial, fungal, and viral infections, including SARS-CoV-2 [16, 17].

In this study the most prominent aetiology of CLD was chronic viral infection (HBV and HCV infections), which was expected considering that the study was conducted at the Infectious Diseases Clinic, where these patients are habitually treated. Patients with NAFLD were frequent too, which was also expected because in the general population this pathology has an average prevalence of 23.7% in Europe [18]. Alcohol abuse–ALD was the most prominent unfavourable prognostic factor regarding the aetiology of CLD. It caused a mortality rate of 60% which was statistically higher compared with other aetiologies. Marjot *et al*. have also identified ALD as a risk factor for a lethal outcome of COVID-19 [6]. This could be partly explained by the fact that ALD is more often diagnosed as advanced liver disease [19]. Furthermore, chronic alcohol abusers are more prone to bacterial infections due to the immunomodulating effect of alcohol itself. The probability of developing acute respiratory distress syndrome (ARDS) is higher in this specific group during sepsis [20, 21]. Results published by Okuno *et al*. must certainly be taken into consideration, whereby patients with ALD had a greater expression of ACE2 receptors (above all on cholangiocytes), the main receptors for initial SARS-CoV-2 binding [22]. Such findings gain in significance having in mind that alcohol abuse has increased significantly in recent years, and this was especially prominent during the COVID-19 pandemic and its unfavourable influence on mental health in the general population [23, 24].

Increased transaminase level prior to COVID-19 were shown to be a negative prognostic factor for a lethal outcome in our study. Increased transaminases are consequences of inflammation and necrosis of hepatocytes. The authors consider that liver necrosis might decrease hepatic reserve and lead to greater propensity to liver damage during COVID-19, either as a direct effect of the virus, immunologically mediated inflammation, or unfavourable liver toxicity of drugs. On the other hand, liver injury affects the choice of drugs used in COVID-19 treatment (such as antivirals, antibiotics, anticoagulation agents, antipyretics etc.). Nevertheless, liver damage influences drug metabolism, as well [25].

Severe pneumonia and respiratory insufficiency with the need for oxygen support in ICU were independent predictors of lethal outcome. This was an expected finding, considering that studies have shown that in patients with CLD, above all cirrhosis, a lethal outcome was most frequently the consequence of respiratory failure. It has been speculated that respiratory insufficiency develops due to pulmonary venous microthrombosis and parenchymal injury, in addition to disrupted haemostatic homeostasis in patients with CLD [5].

Antiviral treatment did not significantly influence the lethal outcome, although there were somewhat fewer deaths in the group that received antivirals (20% vs 25%). This can be explained by the fact that the majority of patients who received antivirals had liver cirrhosis (40%) which by itself, is an unfavourable prognostic factor for COVID-19. Another important reason may be that most of our patients (as many as 75%) received Favipiravir with its questionable influence on lethal outcome among those with COVID-19. Thus, in earlier studies, Favipiravir showed some promising positive results, such as an improvement of the CT pathological findings on the lungs of those stricken or in clearance of SARS-CoV-2 from the nose and throat in these patients [26, 27]. However, in latter studies, that encompassed a higher number of subjects, Favipiravir did not decrease the lethality rate. Thereby, in the meta-analysis conducted by Özlüşen *et al*. Favipiravir did not decrease the lethality rate (OR 1.11, 95% CI 0.64–1.94), nor the requirement for mechanical ventilation (OR 0.50, 95% CI 0.13–1.95) [28]. Even so, the GRADE principles were not used in this large study, and currently an analysis of the influence of Favipiravir on COVID-19 is being conducted by the Cochrane Network and this should bring definitive conclusions [29]. Published data undoubtedly point out the positive effect of corticosteroids and Tocilizumab in patients with severe COVID-19 [30, 31]. Our study failed to demonstrate those positive effect on lethal outcome.

In our study only 20% of subjects were vaccinated for COVID-19. This finding was opsite to the recommendations of the European Association for the Study of the Liver (EASL), which recommends vaccination for persons with CLD, especially if they have advanced liver disease, a liver transplant, hepatobiliary cancer, or they are immunosuppressed [32]. The reason for the low percentage of vaccinated patients in our study lies in part with the inclusion of subjects from March 2020, when the COVID-19 pandemic started in Serbia and vaccines became available only in December 2020. We believe that an even greater reason for such a low rate of vaccination lies in the negative campaigns against vaccination but also in patients’ fear of adverse events and lack of positive advice from healthcare providers. A similar situation was described by Cao *et al*. in patients with cirrhosis [33].

Although increases in transaminases were registered in almost one third of patients, only 3.8% of them experienced deterioration of liver function (decompensation with development of ascites de novo). There are several theories about liver injury during COVID-19, such as the direct cytopathic effect of the SARS-CoV-19 on hepatocytes and biliary epithelial cells via the ACE2 receptors, but also immunological mediated damage. The latter is in connection with a stronger immunological response and the release of cytokines, the toxic effects of drugs on the liver, perturbations of the gut-liver axis, endotheliopathy with subsequent hypoxia and/or ischaemia and hypercoagulability [34]. The assumption is that the same mechanisms could lead to a deterioration of pre-existing liver diseases and elevate the risk of decompensation (acute-on-chronic liver failure—ACLF) [35].

According to our knowledge, this study represents the first analysis of patients with CLDs suffering from COVID-19 to be conducted in Serbia. In contrast to the majority of research carried out so far, this study included etiologically different chronic liver diseases (infectious, metabolic, toxic, autoimmune as well as unknown etiology), and also in different stages (from mild liver fibrosis to terminal stages of the liver disease—decompensated liver cirrhosis, HCC, liver transplantation). Therefore, this is a comprehensive analysis of the impact of chronic liver disease on the course and outcome of COVID-19, and vice versa, the effect of COVID-19 on the exacerbation of CLD is also examined. All of this give significance to and strengthens the value of this study. The authors are certainly aware of the limitations of this study. Firstly, it is a retrospective analysis that was conducted at the height of the current pandemic, and therefore it was unavoidable that certain data would not be available for all subjects (e.g., CT scan score). This shortcoming was overcome by excluding them from the statistical analysis while processing those variables. Considering that the study includes only persons treated in hospital, it is likely that there is reporting bias with over-representation of cases with advanced liver disease or more severe COVID-19. Although 80 subjects with CLD were included in the study, undoubtedly a larger sample would give more strength to the results that were obtained, in particular in respect of the multivariate analysis. The multivariate analysis does not include shortness of breath as a separate variable because its presence almost completely coincided with severe pneumonia. Regardless of these imperfections of the study, the authors believe that none of them has brought its validity into question.

This study demonstrated that severe forms of COVID-19 occur in patients with liver cirrhosis regardless of etiology (as many as 40.7% required treatment in ICU), but also in patients with ALD. These two group of patients suffered a higher mortality rate, compared to others with CLD. On the other hand, worsening of chronic liver disease occured in a third of hospitalized patients with COVID-19.

## Conclusions

Considering that there is still no end in sight for the COVID-19 pandemic, physicians should be aware that liver cirrhosis and severe pneumonia were shown in the study to be independent unfavourable predictors of lethal outcome for persons with CLD infected with the SARS-CoV-2. Furthermore, the rest of the risk factors that univariate statistical analysis showed to be significant predictors of an unfavourable outcome, such as ALD, elevated transaminase values, malignancies as associated comorbidities, and advance age, must not be overlooked. The results obtained in this study will undoubtedly assist with the evaluation of COVID-19 patients who have CLDs and improve their risk stratification, which will ultimately influence the efficiency of treatment and lower mortality.

## Supporting information

S1 File (SAV)
